# Emergency department thoracotomy for the critically injured patient: Objectives, indications, and outcomes

**DOI:** 10.1186/1749-7922-1-4

**Published:** 2006-03-24

**Authors:** C Clay Cothren, Ernest E Moore

**Affiliations:** 1 Department of Surgery, Denver Health Medical Center and the University of Colorado Health Sciences Center, Denver, CO, USA

## Abstract

In the past three decades there has been a significant clinical shift in the performance of emergency department thoracotomy (EDT), from a nearly obligatory procedure before declaring any trauma patient to select patients undergoing EDT. The value of EDT in resuscitation of the patient in profound shock but not yet dead is unquestionable. Its indiscriminate use, however, renders it a low-yield and high-cost procedure. Overall analysis of the available literature indicates that the success of EDT approximates 35% in the patient arriving in shock with a penetrating cardiac wound, and 15% for all penetrating wounds. Conversely, patient outcome is relatively poor when EDT is done for blunt trauma; 2% survival in patients in shock and less than 1% survival with no vital signs. Patients undergoing CPR upon arrival to the emergency department should be stratified based upon injury and transport time to determine the utility of EDT. The optimal application of EDT requires a thorough understanding of its physiologic objectives, technical maneuvers, and the cardiovascular and metabolic consequences.

## Background

The number of patients arriving at hospitals *in extremis*, rather than expiring in the prehospital setting, has increased due to the maturation of regionalized trauma systems throughout the world. Salvage of individuals with imminent cardiac arrest or those already undergoing cardiopulmonary resuscitation often requires immediate thoracotomy as an integral component of their initial resuscitation in the emergency department. The optimal application of emergency department thoracotomy (EDT) requires a thorough understanding of its physiologic objectives, technical maneuvers, and the cardiovascular and metabolic consequences. This review highlights these features and the specific clinical indications of EDT, all of which are essential for the appropriate use of this potentially life-saving yet costly procedure.

### Historical perspective and clinical outcomes

Emergent thoracotomy came into use in the United States for the treatment of heart wounds and anesthesia-induced cardiac arrest in the late 1800's and early 1900's. The concept of a thoracotomy as a resuscitative measure began with Schiff's promulgation of open cardiac massage in 1874 [1] and had expanded indications with treatment of penetrating chest wounds and heart lacerations around the turn of the century [2,3]. With improvement in patient resuscitation and an ongoing evaluation of patient outcomes, the indications for emergent thoracotomy shifted. Initially, cardiovascular collapse from medical causes was the most common reason for thoracotomy in the early 1900's. The demonstrated efficacy of closed-chest compression by Kouwenhoven and colleagues [4] in 1960 and the introduction of external defibrillation in 1965 by Zoll and colleagues [5] virtually eliminated the practice of open-chest resuscitation for medical cardiac arrest. The use of emergent thoracotomy following trauma also declined as less invasive therapeutics, such as pericardiocentesis for cardiac tamponade, were preferred [6]. In the late 1960s, the pendulum toward emergent thoracotomy swung again, as refinements in cardiothoracic surgical techniques salvaged patients with life-threatening chest wounds [7] and use of temporary thoracic aortic occlusion saved patients with exsanguinating abdominal hemorrhage [8,9].

In the past three decades there has been a significant clinical shift in the performance of EDT. While the use of EDT in resuscitation of the patient in profound shock but not yet dead is unquestionable, its indiscriminate use, renders it a low-yield and high-cost procedure [10-12]. During this swing of the pendulum, several groups have attempted to elucidate the clinical guidelines for EDT [13,14]. In 1979, we conducted a critical analysis of 146 consecutive patients undergoing EDT and suggested a selected approach to its use in the moribund trauma patient, based on consideration of the following variables: (1) location and mechanism of injury, (2) signs of life at the scene and on admission to the ED, (3) cardiac electrical activity at thoracotomy, (4) systolic blood pressure response to thoracic aortic cross-clamping [12].

To validate these clinical guidelines, we established a prospective study in which these data were carefully documented in all patients at the time of thoracotomy. In 1982, the first 400 patients were analyzed [11]. A more recent review has summarized the data on 868 patients who have undergone EDT at the Denver Health Medical Center [15]. Of these, 676 (78%) were dead in the ED, 128 (15%) died in the operating room, and 23 (3%) succumbed to multiple organ failure in the surgical intensive care unit. Ultimately, 41 (5%) patients survived, and 34 recovered fully without neurologic sequelae. While this yield may seem low, it is important to emphasize that thoracotomy was done on virtually every trauma patient delivered to the ED. In fact, 624 (72%) were without vital signs in the field, and 708 patients (82%) had no vital signs at the time of presentation to the ED. In contrast, it is equally important to stress that patients without signs of life at the scene but who responded favorably to resuscitation were excluded from this analysis because they did not require EDT; these patients remind the practitioner that prehospital clinical assessments may not always be reliable in triaging these severely injured patients [16]. Indeed, the authors have salvaged a number of individuals sustaining blunt and penetrating trauma who were assessed to have no signs of life at the scene of injury.

The survival rate and percentage of neurologic impairment following EDT varies considerably, due to the heterogeneity of patient populations reported in the literature and the inconsistency of terminology. Clarification of patient physiology and consistency of terms is the first step in standardizing EDT evaluation (Table 1). We have attempted to elucidate the factors affecting survival following EDT by collating data from a number of clinical series reporting on 50 or more patients (Table 2). Objective analysis of these data is limited due to inconsistencies in patient stratification; some reviews provide a specific breakdown of the injury mechanism and clinical status of patients presenting to the ED, while others combine all injury mechanisms. The summarized data confirms EDT has the highest survival rate following isolated cardiac injury; 35% of adult patients presenting in shock, and 20% without vital signs, were salvaged after isolated penetrating injury to the heart if EDT was performed. In contrast, only 1–3% of blunt trauma patients undergoing EDT survive, regardless of clinical status on presentation. Following penetrating torso injuries, 14% of patients requiring EDT are salvaged if they are hypotensive with detectable vital signs, whereas 8% of those who have no vital signs but have signs of life at presentation, and 1% of those without signs of life are salvaged. These findings are reiterated by a recent report incorporating all patients from 24 separate studies undergoing EDT for either blunt or penetrating mechanism [17]; survival rates by mechanism in descending order are 19.4% for isolated cardiac wounds, 16.8% for stab wounds, 4.3% for gunshot wounds, and 1% for blunt trauma.

**Table 1:** Definitions of Terminology Used in Patients Undergoing EDT

emergency department thoracotomy (EDT) = thoracotomy performed in the emergency department for patients arriving *in extremis*; this should not be used interchangeably with nor confused with a thoracotomy that is performed in the operating room (OR) or intensive care unit (ICU) within hours after injury for delayed physiologic deterioration.
"no signs of life" = no detectable blood pressure, respiratory or motor effort, cardiac electrical activity, or pupillary activity (i.e., clinical death).
"no vital signs" = no palpable blood pressure, but demonstrate electrical activity, respiratory effort or pupillary reactivity.

**Table 2:** Survival Following Emergency Department Thoracotomy in Adults

Injury Pattern	Shock	No Vital Signs	No Signs Of Life	Total
*Cardiac*				
Denver (57)	3/9 (33%)	0/7 (0%)	1/53 (2%)	4/69 (6%)
Detroit (58)	9/42 (21%)	3/110 (3%)		12/152 (8%)
Johannesburg (59)				13/108 (12%)
Los Angeles (60)	2/5 (40%)	6/11 (55%)	2/55 (4%)	10/71 (14%)
New York (61)	7/20 (35%)	18/53 (32%)	0/18 (0%)	24/91 (26%)
San Francisco (62)	18/37 (49%)	0/25 (0%)		18/63 (29%)
Seattle (63)	4/11 (36%)	11/47 (23%)		15/58 (26%)
**Overall**	**43/124 (35%)**	**47/254 (19%)**	**4/126 (3%)**	**96/612 (16%)**
*Penetrating*				
Denver (15)	19/78 (24%)	14/399 (4%)		33/477 (7%)
Detroit (58)	9/42 (21%)	3/110 (3%)		12/152 (8%)
Houston (64)	14/156 (9%)	18/162 (11%)		32/318 (10%)
Indianapolis (65)	3/7 (43%)	1/50 (2%)	0/80 (0%)	4/137 (3%)
Johannesburg (59)	31/413 (8%)	10/149 (7%)	1/108 (1%)	42/670 (6%)
Los Angeles (60)	2/5 (40%)	6/11 (55%)	2/55 (4%)	10/71 (14%)
New York (66)	8/32 (25%)	8/77 (10%)	0/25 (0%)	16/134 (12%)
Oakland (67)	8/24 (33%)		2/228 (1%)	10/252 (4%)
San Francisco (62)				32/198 (30%)
Seattle (63)	4/11 (36%)	11/47 (23%)		15/58 (25%)
Washington (68)	7/13 (54%)	3/47 (6%)		10/60 (17%)
**Overall**	**145/1007 (14%)**	**100/1252 (8%)**	**6/615 (1%)**	**283/2986 (10%)**
*Blunt*				
Denver (15)	4/86 (5%)	4/311 (1%)		8/397 (2%)
Houston (64)	0/42 (0%)	0/27 (0%)		0/69 (0%)
Johannesburg (59)	1/109 (1%)	0/39 (0%)	0/28 (0%)	1/176 (1%)
San Francisco (62)				1/60 (2%)
Seattle (63)				1/88 (1%)
**Overall**	**5/237 (2%)**	**4/377 (1%)**	**0/28 (0%)**	**11/790 (1.4%)**

Emerging data indicates the clinical results in the pediatric population mirror that of the adult experience. One might expect that children would have a more favorable outcome compared to adults; however, this has not been borne out in multiple studies [18-22]. Beaver and colleagues reported no survivors among 27 patients, from 15 months to 14 years of age, undergoing postinjury EDT at Johns Hopkins Hospital [18]. Powell and coworkers, described an overall survival of 20% (3 of 15 patients) in patients ranging from 4 to 18 years [21]. In a study at Denver Health Medical Center, encompassing an 11-year experience with 689 consecutive EDT, we identified 83 patients (12%) who were under 18 years old [19]. Survival by injury mechanism was 9% (1 of 11) for stab wounds, 4% (1 of 25) for gunshot wounds, and 2% (1 of 47 patients) for blunt trauma. Among 69 patients presenting to the ED without vital signs, only 1 patient (1%) survived (with a stab wound). This contrasted to a salvage of 2 (14%) among 14 patients with vital signs. The outcome in blunt trauma, the predominant mechanism of lethal injury in children, was disappointing, with only 2% salvage, and no survivors when vital signs were absent. Thus, as in adults, outcome following EDT in the pediatric population is largely determined by injury mechanism and physiologic status on presentation to the emergency department.

There is disagreement about the use of EDT in the patient population undergoing cardiopulmonary resuscitation prior to arrival in the emergency department. Although there have been multiple reports with low survival rates and dismal outcomes following prehospital CPR, termination of resuscitation in the field should not be performed in all patients [23]. Our most recent evaluation, incorporating 26 years of experience, indicates EDT plays a significant role in the critically injured patient undergoing prehospital CPR [13]. We believe this study provides simple, clear guidelines for the use of EDT as a resuscitative measure to ensure that all potentially salvageable patients are included (Table 3).

**Table 3:** Current indications and contraindications for EDT

Indications:
Salvageable postinjury cardiac arrest:
Patients sustaining witnessed penetrating trauma with < 15 minutes of prehospital CPR.
Patients sustaining witnessed blunt trauma with < 5 minutes of prehospital CPR.
Persistent severe postinjury hypotension (SBP ≤ 60 mmHg) due to:
Cardiac tamponade
Hemorrhage – intrathoracic, intraabdominal, extremity, cervical
Air embolism
Contraindications:
Penetrating trauma: CPR > 15 minutes and no signs of life (pupillary response, respiratory effort, or motor activity)
Blunt trauma: CPR > 5 minutes and no signs of life or asystole

In sum, overall analysis of the available literature indicates that the success of EDT approximates 35% in the patient arriving in shock with a penetrating cardiac wound, and 15% for all penetrating wounds. Patients undergoing CPR upon arrival to the emergency department should be stratified based upon injury and transport time to determine the utility of EDT. Conversely, patient outcome is relatively poor when EDT is done for blunt trauma; 2% survival in patients in shock and less than 1% survival with no vital signs.

### Indications for EDT

**Figure 1:**
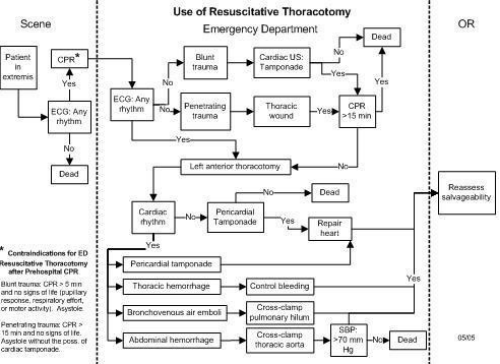
Algorithm directing the use of EDT in the multiply injured trauma patient.

Based on our 26 successive years of EDT prospective analysis, we propose current indications for EDT [13] (Table 3). Our current decision algorithm for resuscitation of the moribund trauma patient and use of EDT was formulated and implemented as a key clinical pathway in the emergency department (Figure 1). At the scene, patients in extremis without electrical cardiac activity are declared dead. Patients in extremis but with electrical cardiac activity are intubated, supported with cardiac compression, and rapidly transported to the ED.

On arrival to the ED, the time from initiation of CPR is recorded; blunt trauma patient with greater than 5 minutes of prehospital CPR and no signs of life are declared, while penetrating trauma patients with greater than 15 minutes of prehospital CPR and no signs of life are pronounced. Patients within the time guidelines or those with signs of life trigger ongoing resuscitation and EDT. After performing a left anterior thoracotomy and pericardotomy, the patient's intrinsic cardiac activity is evaluated; patients in asystole without associated cardiac injury are declared. Patient's with a cardiac wound, tamponade, and associated asystole are aggressively treated; the cardiac wound is repaired first followed by manual cardiac compressions and intracardiac injection of epinephrine. Following several minutes of such treatment and volume resuscitation, one should reassess salvageability; we define this as the patient's ability to generate a systolic blood pressure > 70 mmHg.

Patients with an intrinsic rhythm following EDT should be treated according to underlying pathology. Those with tamponade should undergo cardiac repair, either in the trauma bay or the operating room. Control of intrathoracic hemorrhage may entail hilar crossclamping, digital occlusion of the direct injury, or even packing of the apices for subclavian injuries. Treatment of bronchovenous air embolism includes crossclamping of the hilum, putting the patient in Trendelenberg's position, aspirating the left ventricle and aortic root, and massaging the coronaries. Finally, aortic crossclamping is performed to decrease the required effective circulating volume, for either thoracic or abdominal sources of hemorrhage, and facilitate resuscitation. In all of these scenarios, reassessment of the patient following intervention and aggressive resuscitation efforts is performed, with the goal systolic pressure of 70 mmHg used to define salvageability.

### Physiologic rationale

The primary objectives of EDT are to (a) release pericardial tamponade; (b) control cardiac hemorrhage; (c) control intrathoracic bleeding; (d) evacuate massive air embolism; (e) perform open cardiac massage; and (f) temporarily occlude the descending thoracic aorta. Combined, these objectives attempt to address the primary issue of cardiovascular collapse from mechanical sources or extreme hypovolemia.

### Release pericardial tamponade and control cardiac hemorrhage

The highest survival rate following EDT is in patients with penetrating cardiac wounds, especially when associated with pericardial tamponade [7,17]. Early recognition of cardiac tamponade, prompt pericardial decompression, and control of cardiac hemorrhage are the key components to successful EDT and patient survival following penetrating wounds to the heart [24]. The egress of blood from the injured heart, regardless of mechanism, results in tamponade physiology. The classic description of clinical findings, Beck's triad, is rarely observed in the emergency department; therefore, a high index of suspicion in the at-risk patient sustaining penetrating torso trauma is crucial, with prompt intervention essential. The first two phases of cardiac tamponade – restricted ventricular diastolic filling, compromised stroke volume and coronary perfusion, and diminished cardiac output – may be aggressively managed with definitive airway control, volume resuscitation to increase preload, and pericardiocentesis. The patient in the third phase of tamponade – intrapericardial pressure approaches the ventricular filling pressure with profound hypotension (SBP < 60) – should undergo EDT rather than pericardiocentesis as the management for evacuation of pericardial blood [25,26]. Following release of tamponade, the source of tamponade can be directly controlled with appropriate interventions based on the underlying injury.

### Control intrathoracic hemorrhage and perform open cardiac massage

Life-threatening intrathoracic hemorrhage occurs in less than 5% of patients following penetrating injury presenting to the ED, and in even lower percentage of patients sustaining blunt trauma [27]. The most common injuries include penetrating wounds to the pulmonary hilum and great vessels; less commonly seen are torn descending thoracic aortic injuries with frank rupture or penetrating cardiac wounds exsanguinating into the thorax through a traumatic pericardial window. There is a high mortality rate in injuries to the pulmonary or thoracic great-vessel lacerations due to the lack of hemorrhage containment by adjacent tissue tamponade or vessel spasm. Either hemithorax can rapidly accommodate more than half of a patient's total blood volume before overt physical signs of hemorrhagic shock occur. Patients with exsanguinating wounds require EDT with rapid control of the source of hemorrhage if they are to be salvaged.

In patients with cardiopulmonary arrest, external chest compression provides approximately 20 to 25% of baseline cardiac output, with 10 to 20% of normal cerebral perfusion [28-30]. While this degree of vital organ perfusion can provide reasonable salvage rates for 15 minutes, few normothermic patients survive 30 minutes of closed-chest compression. Moreover, in models of inadequate intravascular volume (hypovolemic shock) or restricted ventricular filling (pericardial tamponade), external chest compression fails to augment arterial pressure or provide adequate systemic perfusion; the associated low diastolic volume and pressure result in inadequate coronary perfusion [31]. Therefore, closed cardiac massage is ineffective for postinjury cardiopulmonary arrest. The only potential to salvage the injured patient with ineffective circulatory status is immediate EDT.

### Achieve thoracic aortic cross clamping

The rationale for temporary thoracic aortic occlusion in the patient with massive hemorrhage is two-fold. First, in patients with hemorrhagic shock, aortic cross clamping redistributes the patient's limited blood volume to the myocardium and brain [9]. Second, patients sustaining intraabdominal injury may benefit from aortic cross clamping due to reduction in subdiaphragmatic blood loss [8]. Temporary thoracic aortic occlusion augments aortic diastolic and carotid systolic blood pressure, enhancing coronary as well as cerebral perfusion [32,33]. Experimental observations suggest that temporary aortic occlusion is valuable in the patient either with shock due to non-thoracic trauma or in patients with continued shock following the repair of cardiac or other exsanguinating wounds [34,35]. Indeed, occlusion of the descending thoracic aorta appears to increase the return of spontaneous circulation following cardiopulmonary resuscitation [36,37]. Reports of successful resuscitation using EDT in patients in hemorrhagic shock and even sustaining cardiac arrest following extremity and cervical injuries exist [38]. In these situations, EDT may be a temporizing measure until the patient's circulating blood volume can be replaced by blood product transfusion. In carefully selected patients, aortic cross clamping may effectively redistribute the patient's blood volume until external replacement and control of the hemorrhagic source is possible. However, once the patient's blood volume has been restored, the aortic cross clamp should be removed, as there is substantial metabolic cost and a finite risk of paraplegia associated with the procedure [39-41]. Typically, complete removal of the aortic cross clamp or replacement of the clamp below the renal vessel should be performed within 30 minutes.

### Evacuate bronchovenous air embolism

Bronchovenous air embolism can be a subtle entity following thoracic trauma, and is likely to be much more common than is recognized [42-44]. The clinical scenario typically involves a patient sustaining penetrating chest injury who precipitously develops profound hypotension or cardiac arrest following endotracheal intubation and positive-pressure ventilation. Traumatic alveolovenous communications produce air emboli that migrate to the coronary arterial systems; any impedance in coronary blood flow causes global myocardial ischemia and resultant shock. The production of air emboli is enhanced by the underlying physiology – there is relatively low intrinsic pulmonary venous pressure due to associated intrathoracic blood loss and high bronchoalveolar pressure from assisted positive pressure ventilation. This combination increases the gradient for air transfer across bronchovenous channels [45]. Although more often observed in penetrating trauma, a similar process may occur in patients with blunt lacerations of the lung parenchyma.

Immediate thoracotomy with pulmonary hilar cross clamping prevents further propagation of pulmonary venous air embolism. Thoracotomy with opening of the pericardium also provides access to the cardiac ventricles; with the patient in the Trendelenburg's position (done to trap air in the apex of the ventricle), needle aspiration is performed to remove air from the cardiac chambers. Additionally, vigorous cardiac massage may promote dissolution of air already present in the coronary arteries [44]. Aspiration of the aortic root is done to alleviate any accumulated air pocket, and direct needle aspiration of the right coronary artery may be lifesaving.

### Technical pearls

The optimal benefit of EDT is achieved by a surgeon experienced in the management of intrathoracic injuries. The emergency physician, however, should not hesitate to perform the procedure in the moribund patient with a penetrating chest wound when thoracotomy is the only means of salvage. The technical skills needed to perform the procedure include the ability to perform a rapid thoracotomy, pericardiotomy, cardiorrhaphy, and thoracic aortic cross clamping; familiarity with vascular repair techniques and control of the pulmonary hilum are advantageous. As the procedure has been described in detail elsewhere, we will briefly touch on some "pearls" which may facilitate one's approach and success in performing EDT.

### Thoracic incision

**Figure 2:**
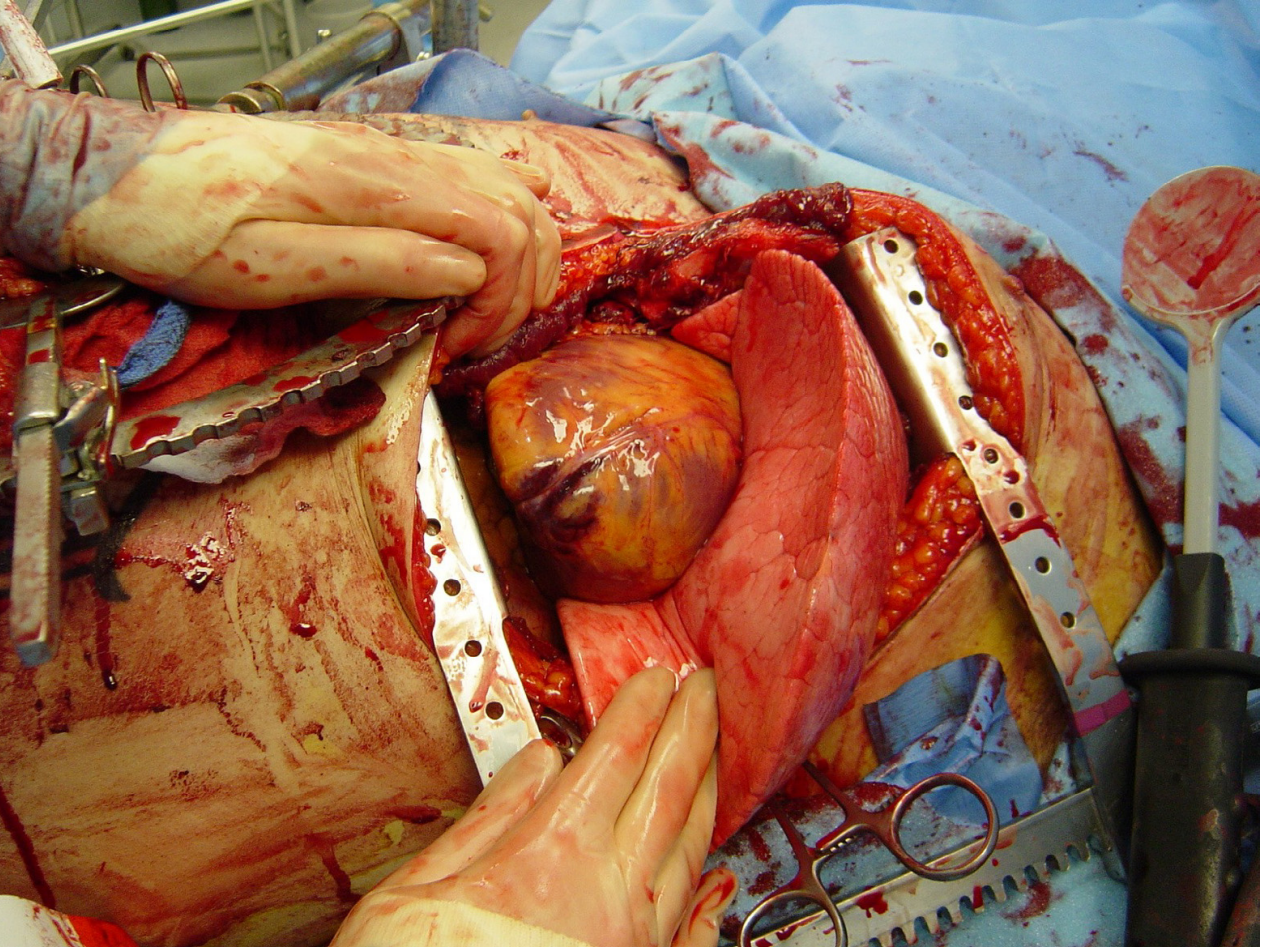
A generous thoracotomy incision is performed through the fourth or fifth intercostal space; the incision should start to the right of the sternum, and begin curving into the axilla at the level of the left nipple. The Finochiettos' rib retractor should be placed with the handle directed inferiorly toward the bed, in case transverse sternal split is warranted.

Upon patient arrival and determination of the need for EDT, the patient's left arm should be placed above the head to provide unimpeded access to the left chest. The thoracotomy incision should start on the right side of the sternum; if sternal transection is required, this saves the time consuming step of performing an additional skin incision (Figure 2). As the initial incision is carried transversely across the chest, as one passes beneath the nipple a gentle curve in the incision toward the patient's axilla rather than direct extension to the bed should be performed; this curvature in the skin correlates with the natural curvature of the rib cage. The initial execution of a clamshell thoracotomy should be done in hypotensive patients with penetrating wounds to the right chest. This provides immediate, direct access to a right-sided pulmonary or vascular injury while still allowing access to the pericardium from the left side for open cardiac massage. Clamshell thoracotomy may also be considered in patients with presumed air embolism, providing access to the cardiac chambers for aspiration, coronary vessels for massage, and bilateral lungs for obliteration of the source. Once the right pleural space is opened, the rib retractor should be moved to more of a midline position to enhance separation of the chest wall for maximal exposure. When visualization of penetrating wounds in the aortic arch or major branches is needed, the superior sternum is additionally split in the midline. If the sternum is divided transversely, the internal mammary vessels must be ligated when perfusion is restored; this may be performed using either a figure of eight suture with 2-0 silk or by clamping the vessel with a tonsil and individually ligating it with a 2-0 silk tie.

### Pericardiotomy and cardiac hemorrhage control

**Figure 3:**
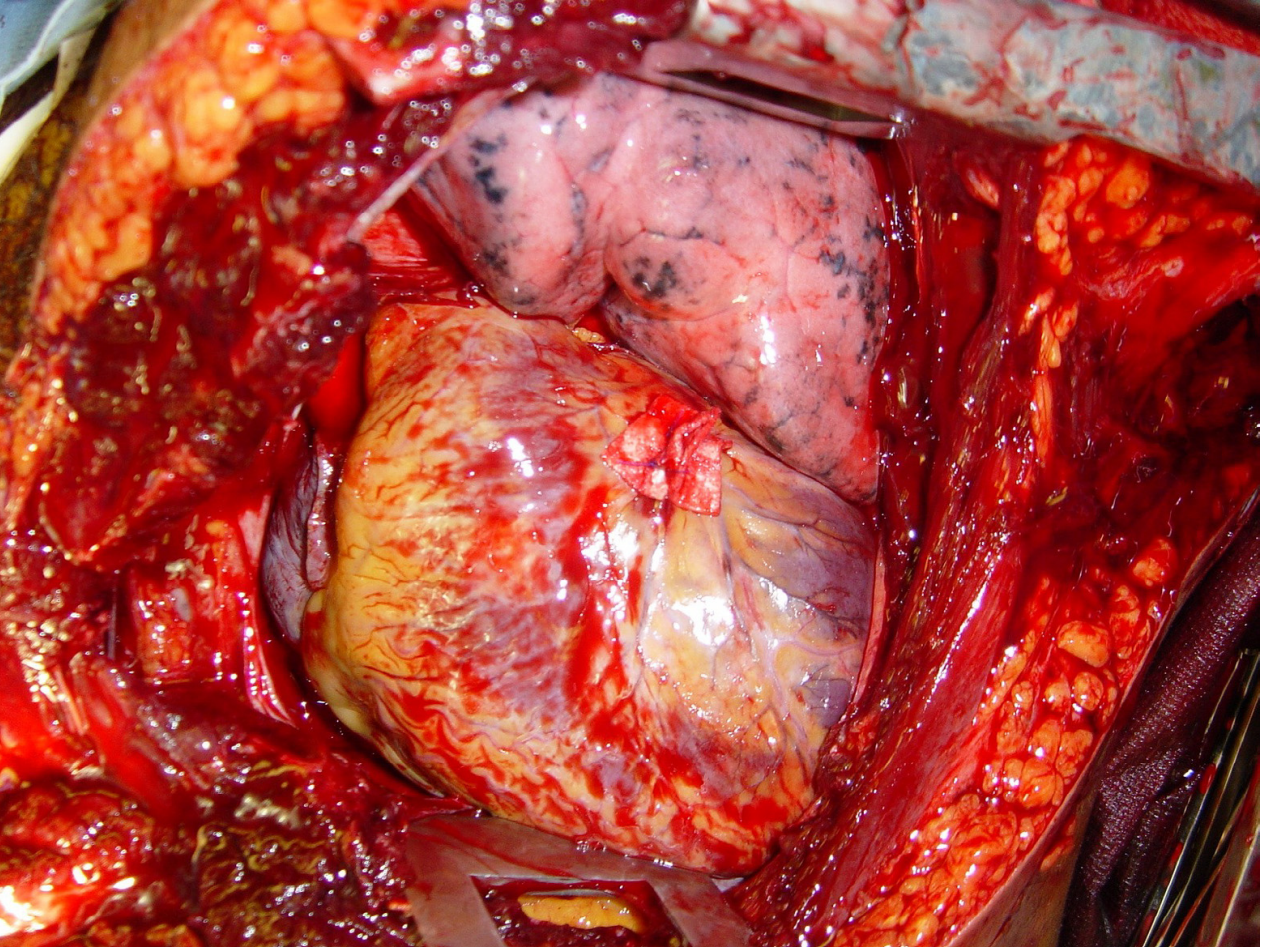
Cardiorrhaphy of the right ventricle is buttressed with pledgets; ligation of a coronary artery can be avoided by performing vertical mattress sutures.

If the pericardium is not tense with blood it may be picked up at the apex with toothed forceps and sharply opened with scissors. If tense pericardial tamponade exists, a knife or the sharp point of a scissors is often required to initiate the pericardiotomy incision. Prompt hemorrhage control is paramount for a cardiac injury. In the beating heart, cardiac bleeding sites should be controlled immediately with digital pressure on the surface of the ventricle and partially occluding vascular clamps on the atrium or great vessels. Efforts at definitive cardiorrhaphy may be delayed until initial resuscitative measures have been completed. In the nonbeating heart, cardiac repair is done prior to defibrillation and cardiac massage. Cardiac wounds in the thick walled left ventricle are best repaired with 3-0 non-absorbable running or horizontal mattress sutures. Buttressing the suture repair with Teflon pledgets is ideal for the thinner right ventricle. When suturing a ventricular laceration, care must be taken not to incorporate a coronary vessel into the repair. In these instances, vertical mattress sutures should be used to exclude the coronary and prevent cardiac ischemia (Figure 3). In the more muscular left ventricle, particularly with a linear stab wound, control of bleeding can often be temporized with a skin-stapling device. Low-pressure venous, atrial, and atrial appendage lacerations can be repaired with simple running or pursestring sutures. Use of a foley catheter for temporary occlusion of cardiac injuries has been suggested; in our experience this may inadvertently extend the injury due to traction forces.

### Advanced cardiac life support interventions including cardiac massage

**Figure 4:**
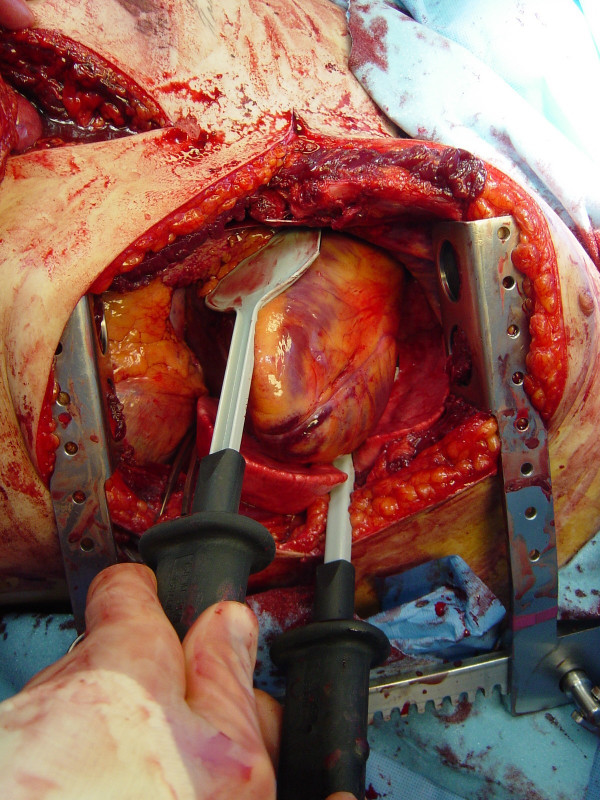
Internal paddles for defibrillation are positioned on the anterior and posterior aspects of the heart.

The restoration of organ and tissue perfusion may be facilitated by a number of interventions. Dysrhythmias should be treated according to current guidelines [46] and internal defibrillation has similar indications as closed chest CPR. Familiarity with the internal cardiac paddles and appropriate charging dosages in joules is required (Figure 4). In the event of cardiac arrest, bimanual internal massage of the heart should be instituted promptly. We prefer to do this with a hinged clapping motion of the hands, with the wrists apposed, sequentially closing from palms to fingers. The ventricular compression should proceed from the cardiac apex to the base of the heart. The two-handed technique is strongly recommended, as the one-handed massage technique poses the risk of myocardial perforation with the thumb. Pharmocologic adjuncts to increase coronary and cerebral perfusion pressure may be needed; the first agent in resuscitation at this juncture is intracardiac epinephrine. Epinephrine should be administered using a specialized syringe, which resembles a spinal needle, directly into the left ventricle. Typically the heart is lifted up slightly to expose the more posterior left ventricle, and care is taken to avoid the circumflex coronary during injection.

### Thoracic aortic occlusion

**Figure 5:**
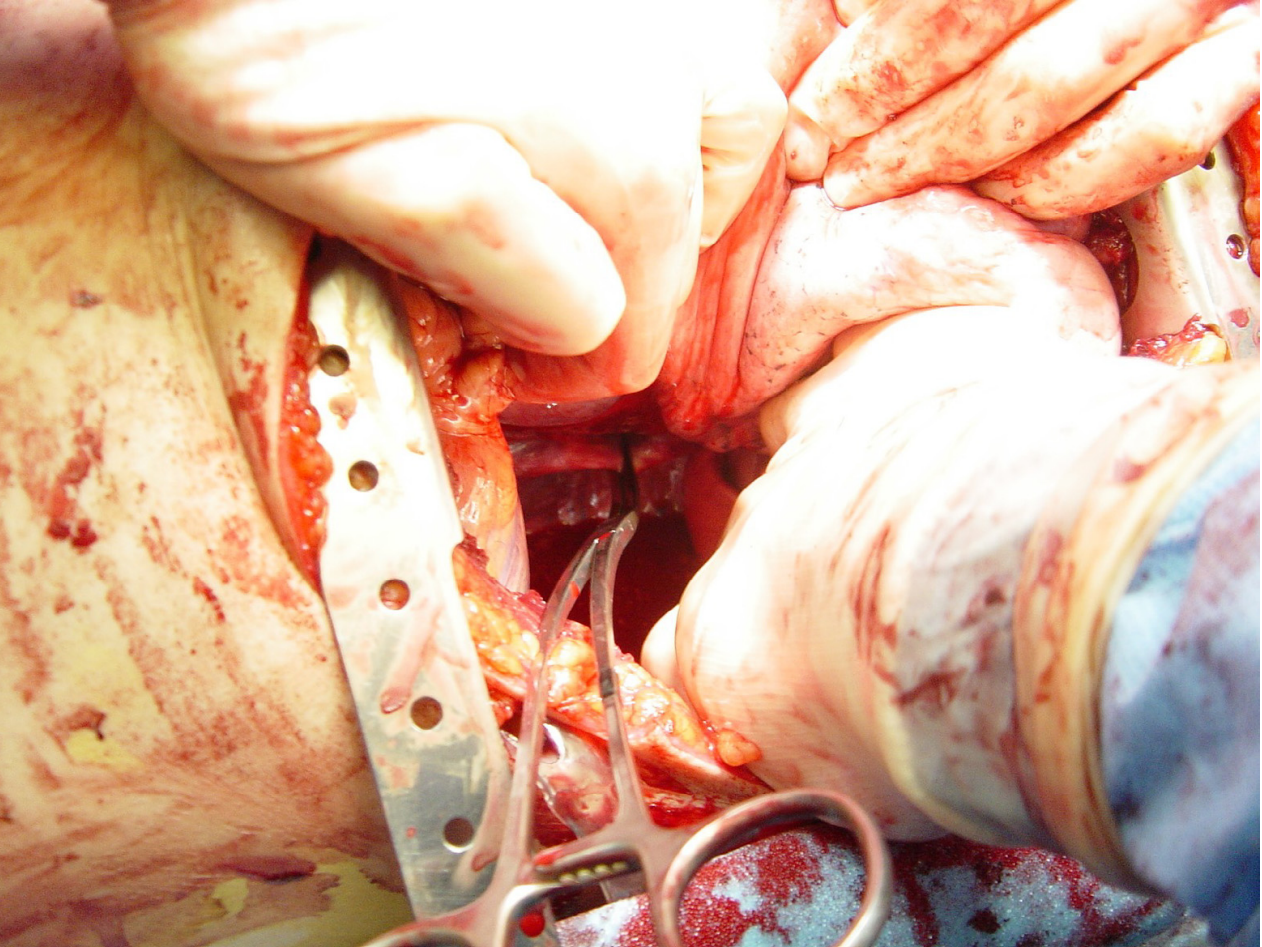
Aortic crossclamp is applied with the left lung retracted superiorly, below the inferior pulmonary ligament, just above the diaphragm. The flaccid aorta is identified as the first structure encountered on top of the spine when approached from the left chest.

If hypotension persists (SBP < 70 mmHg) following thoracotomy and pericardiotomy, the descending thoracic aorta should be occluded to maximize coronary perfusion. We prefer to cross-clamp the thoracic aorta inferior to the left pulmonary hilum (Figure 5). Although some advocate taking down the inferior pulmonary ligament to better mobilize the lung, this is unnecessary and risks injury to the inferior pulmonary vein. Dissection of the thoracic aorta is optimally performed under direct vision by incising the mediastinal pleura and bluntly separating the aorta from the esophagus anteriorly and from the prevertebral fascia posteriorly; if excessive hemorrhage limits direct visualization, which is the more realistic clinical scenario, blunt dissection with one's thumb and fingertips can be done to isolate the descending aorta. If the aorta cannot be easily isolated from the surrounding tissue, digitally occlude the aorta against the spine to affect aortic occlusion. Although occlusion of the thoracic aorta is typically performed after pericardiotomy, this may be the first maneuver upon entry into the chest in patients sustaining extrathoracic injury and associated major blood loss.

### Physiologic consequences and optimizing oxygen transport

Once life-threatening intrathoracic injuries are controlled or temporized, the major challenge is restoring the patient's hemodynamic integrity and minimizing vital-organ reperfusion injury. Aortic cross clamping may be life saving during acute resuscitation, but there is a cost to the patient. Occlusion of the aorta results in a reduction in abdominal visceral blood flow to 2 to 8% of baseline values [41,42]; this decrease in visceral blood flow magnifies the metabolic cost of shock, results in tissue acidosis and increased oxygen debt, and may ultimately contribute to post-ischemic multiple organ failure [42]. Additionally, return of aortic flow may not result in normalization of flow to vital organs; in animal models, blood flow to the kidneys remained at 50% of baseline despite a normal cardiac output [42]. The metabolic penalty of aortic cross clamping becomes exponential when the normothermia occlusion time exceeds 30 minutes [47-49]. Hypoxia of distal organs induces the elaboration, expression, and activation of inflammatory cell adhesion molecules and inflammatory mediators; this systemic inflammatory response syndrome (SIRS) has been linked to impaired pulmonary function and multiple organ failure [50]. Consequently, the aortic clamp should be removed as soon as effective cardiac function and adequate systemic arterial pressure have been achieved.

Removal of aortic occlusion may result in further hemodynamic sequelae [51]. Aside from the abrupt reperfusion of the ischemic distal torso, and washout of metabolic products and inflammatory mediators associated with aortic declamping, there are direct effects on the cardiopulmonary system. Thoracic cross clamping in the normovolemic patient may be deleterious because of increased myocardial oxygen demands resulting from the increased systemic vascular resistance [51]. The return of large volumes of blood from the ischemic extremities, with its lower pH, elevated lactate, and other mediators may exert a cardiodepressant activity on myocardial contractility [52]. Overzealous volume loading during aortic occlusion may also result in left ventricular strain, acute atrial and ventricular dilatation and, consequently, precipitous cardiac failure [42]. Following release of aortic occlusion there is impaired left ventricular function, systemic oxygen utilization, and coronary perfusion pressure in the postresuscitation period [51,53]. The transient fall in coronary perfusion may not be clinically relevant in patients with efficient coronary autoregulation; however, in patients with coronary disease or underlying myocardial hypertrophy, this increase in cardiac work may results in clinically critical ischemia [53].

Following EDT, patients are frequently in a tenuous physiologic state. The combination of direct cardiac injury, ischemic myocardial insult, myocardial depressants, and pulmonary hypertension adversely impact postinjury cardiac function. Additionally, aortic occlusion induces profound anaerobic metabolism, secondary lactic academia, and release of other reperfusion-induced mediators. Consequently, once vital signs return, the resuscitation priorities shift to optimizing cardiac function and maximizing oxygen delivery to the tissues. The ultimate goal of resuscitation is adequate tissue oxygen delivery and cellular oxygen consumption. Circulating blood-volume status is maintained at the optimal level of cardiac filling in order to optimize cardiac contractility, and the oxygen-carrying capacity of the blood is maximized by keeping the hemoglobin above 7–10 g/dL. If these measures fail to meet resuscitative goals [54] (e.g., oxygen delivery ≥500 mL/min/^2^, resolution of base deficit, or clearance of serum lactate), inotropic agents are added to enhance myocardial function.

### Complications of EDT

Technical complications of EDT involve virtually every intrathoracic structure. The list of such misadventures included lacerations of the heart, coronary arteries, aorta, phrenic nerves, esophagus, and lungs, as well as avulsion of aortic branches to components of the mediastinum. Additional postoperative morbidity among ultimate survivors of EDT includes recurrent chest bleeding, infection of the pericardium, pleural spaces, sternum, and chest wall, and post-pericardiotomy syndrome. Previous thoracotomy, typically seen in patients following coronary bypass, virtually assures technical problems from the presence of dense pleural adhesions and is therefore a relative contraindication to EDT.

There is a finite risk to the health care providers and trauma team performing an EDT [55]. The use of EDT by necessity involves the rapid use of sharp surgical instruments and exposure to the patient's blood. Even during elective procedures in the OR, the contact rate of patient's blood with the surgeon's skin can be as high as 50%, and the contact rate of patients' blood with health care workers' blood as high as 60%. The overall seroprevalence rate of human immunodeficiency virus (HIV) among patients admitted to the ED for trauma is around 4%, but is much higher among the subgroup of patients most likely to require an EDT, e.g., 14% of penetrating trauma victims and nearly 30% of intravenous drug abusers. Caplan and colleagues [56] found that 26% of acutely injured patients had evidence of exposure to HIV (4%), hepatitis B (20%), or hepatitisC virus (14%); there was no difference in the incidence comparing blunt to penetrating trauma. Thus, the likelihood of a health care worker sustaining exposure to HIV or hepatitis in the ED is substantial.

## Conclusion

As clinicians faced with increasing scrutiny over appropriation of resources, it is critical to identify the patient population who will benefit from EDT. Resuscitative efforts should not be abandoned prematurely in the potentially salvageable patient but field assessment of salvageability is unreliable. The proposed algorithm clearly defines the indications for EDT in the current era. Our clinical pathway attempts to optimize resource utilization, but outcomes must continue to be evaluated, searching for more definitive predictors of neurologic outcome. Use of more advanced monitoring devices in the ED, together with further elucidation of the characteristics of irreversible shock, may permit a more physiologic prediction of outcome for these critically injured patients in the future.
